# Dietary factors associated with lifetime asthma or hayfever diagnosis in Australian middle-aged and older adults: a cross-sectional study

**DOI:** 10.1186/1475-2891-11-84

**Published:** 2012-10-12

**Authors:** Richard R Rosenkranz, Sara K Rosenkranz, Kelly JJ Neessen

**Affiliations:** 1 School of Science and Health, University of Western Sydney, Campbelltown, NSW, Australia; 2 Department of General Practice, University of Western Sydney, Campbelltown, NSW, Australia; 3 Department of Human Nutrition, Kansas State University, Manhattan, KS, USA; 4 Department of Human Nutrition, Wageningen University, Wageningen, Netherlands

**Keywords:** Asthma, Hayfever, Airway health, Diet, Western diet, Meat, Adult, Older adult

## Abstract

**Background:**

There is abundant research relevant to genetic and environmental influences on asthma and hayfever, but little is known about dietary risk factors in Australian adults. This study’s purpose was to identify dietary factors associated with lifetime asthma (AS) and asthma or hayfever (AS/HF) diagnosis in Australian middle-aged and older adults.

**Methods:**

From The 45 and Up Study baseline self-report data, this study included 156,035 adult men and women. Participants were sampled from the general population of New South Wales, Australia in 2006–2009. About 12% of participants reported ever receiving an AS diagnosis (men 10%; women 14%) and 23% reported AS/HF diagnosis (men 19%; women 26%). Following principle components factor analysis, dietary items loaded onto one of four factors for men (meats/cheese; fruits/vegetables; poultry/seafood; grains/alcohol) or five factors for women (meats; fruits/vegetables; poultry/seafood; cereal/alcohol; brown bread/cheese). Logistic regression was used to analyze the associations between dietary factors and AS or AS/HF diagnosis.

**Results:**

For men, the meats/cheese factor was positively associated with AS (AOR = adjusted odds ratio for highest versus lowest quintile = 1.18, 95%CI = 1.08, 1.28; *P*_trend_ = 0.001) and AS/HF (AOR for highest versus lowest quintile = 1.22, 95%CI = 1.14, 1.29; *P*_trend_ < 0.001). Poultry/seafood was also associated with AS/HF in men (AOR for highest versus lowest quintile = 1.11, 95%CI = 1.04, 1.17; *P*_trend_ = 0.002). For women, significant risk factors for AS/HF included meats (AOR for highest versus lowest quintile = 1.25, 95%CI = 1.19, 1.31; *P*_trend_ = 0.001), poultry/seafood (AOR for highest versus lowest quintile = 1.06, 95%CI = 1.01, 1.12; *P*_trend_ = 0.016), and fruits/vegetables (AOR for highest versus lowest quintile = 1.07, 95%CI = 1.02, 1.12; *P*_trend_ = 0.011). In contrast, the cheese/brown bread dietary factor was protective against AS in women (AOR for highest versus lowest quintile = 0.88, 95%CI = 0.82, 0.94; *P*_trend_ < 0.001).

**Conclusions:**

Generally, diets marked by greater intakes of meats, poultry, and seafood were associated with diagnosed AS and AS/HF. Taken together, these findings suggest that adherence to a more meat-based diet may pose risk for AS and AS/HF in Australian adults.

## Background

Asthma is a chronic inflammatory disorder characterized by increased responsiveness to environmental triggers and narrowing of the airways, which may lead to wheezing, breathlessness, chest tightness, and coughing [1]. Asthmatics are more likely to suffer from allergic conditions such as eczema and hayfever, and are also more likely to have diabetes, arthritis, heart disease, stroke, cancer and osteoporosis [2-4]. Having hayfever in childhood is a strong predictor of asthma onset later in life [5-7]. This association might be explained by atopic sensitization, a shared link with immunoglobulin E sensitization to common environmental allergens [5].

### Prevalence of asthma and hayfever

About 300 million people worldwide have asthma, and around 400 million are thought to have hayfever [8]. Around 70-80% of people with asthma also report having hayfever, and up to 40% of those with hayfever also have asthma [6,8]. Prevalence of these conditions has increased over recent decades, and the highest rates are seen in Westernized and developed countries [4,8,9]. Asthma and hayfever each pose a burden to public health resources, contribute to poor quality of life, disability, and death [9]. Every year, about 250,000 deaths are attributable to asthma worldwide, and asthma is the fifth leading cause of death in developed countries [4,10]. Asthma incidence is possible throughout life, and most asthma-related deaths occur in older adults [11]. Australia leads the world in prevalence of Asthma [9], but reasons for the relatively high prevalence of asthma in the Australian population are unclear [2].

### Influences on asthma

Lifestyle, environmental and genetic factors may influence risk of asthma independently or interactively [2,12,13]. Triggers of asthma-related symptoms include chemical irritants, viral infections, air pollutants and allergens [2]. Evidence suggests that recent increases in asthma prevalence are due to change in environment and lifestyle, rather than change in genotype [12,13]. Lifestyle factors such as obesity, physical activity, and dietary habits have been shown to influence the development of asthma [14,15].

### Diet and asthma

A review of mainly observational studies of children suggests that increased risk of asthma is associated with low intake of fruits and vegetables, fish, butter and dairy fat, vitamin C and E, beta-carotene, selenium, magnesium, and omega–3 fatty acids, and a high intake of sodium, margarine and omega–6 fatty acids [16]. Other studies of children have shown an inverse relationship between adherence to a Mediterranean diet (characterized by high intakes of fruits and vegetables, nuts, whole grains, unsaturated fatty acids, and fish, combined with low meat consumption and moderate dairy intake) and prevalence of asthma [17-19], although not all studies have shown this relationship [20]. Diets rich in starch, cereals, and vegetables have shown a protective association with asthma [21] and allergic rhino conjunctivitis [22]. Evidence also suggests that foods and diets high in fat are frequently associated with asthma [21,23-25], rhinitis [26,27], allergy, and respiratory health [28], but some studies have not found these relationships [12].

In studies of diet and adult asthma, results are less clear [29,30]. There is evidence, however, that a high margarine intake increases the risk of asthma in adulthood [31]. The consumption of fish may be important in preventing onset of adult asthma [32,33]. Molinas and colleagues found that fish consumption at least once a month could protect against diseases such as asthma and allergic rhino conjunctivitis [32].

Recently, studies in nutritional epidemiology have shifted the focus from individual foods or nutrients toward dietary patterns, which provide greater context for dietary exposure [29,30,34-37]. One such study on adult asthma showed a non-significant relationship between a “vegetarian” dietary pattern and asthma [30]. A recent large prospective study found no clear relationship between three dietary patterns (termed “nuts and wine,” “prudent,” and “Western” patterns) and asthma [36]. In that study, only a decreased risk of frequent asthma attacks was found for the “nuts and wine” pattern [36]. Other studies reflect the possible contribution of a Western diet (characterized by high intakes of red meat, saturated fat, and refined grains, combined with low intakes of whole grains, fresh fruits and vegetables) to the increase in asthma [21,23], supporting other studies showing that a diet high in various fruits and vegetables may have a protective association with allergic rhinitis [38] and asthma in adulthood [33,39].

In a study of asthmatic adults, adherence to a Mediterranean diet increased the likelihood that asthma was well controlled [40]. Results from a cross-sectional study in the Netherlands, however suggest that a cosmopolitan diet (characterized by higher intakes of vegetables, fish and chicken) was associated with increased risk of wheeze and asthma, while a traditional Dutch diet (characterized by a high intake of meat and potatoes and lower intake of soy and cereal) was a risk factor for impaired lung function [35].

In the extant literature, there is little overall consensus on dietary risk or protective factors for asthma in adults, particularly for studies assessing dietary factors [1]. There is also scant evidence available regarding Australian adults, a heterogeneous population, and there is a dearth of studies addressing diet and hayfever. Therefore, the purpose of this study was to identify associations between dietary factors and lifetime prevalence of asthma or asthma/hayfever in Australian middle-aged and older adults.

## Methods

### Subjects

The 45 and Up Study is a large prospective study of men and women aged 45 and over who reside in New South Wales, Australia [41]. From February 2006 to April 2009, participants were recruited from the Medicare Australia enrollment database (18% response rate), with oversampling of rural residents and individuals aged 80 years and over. The present study included data from 156,035 participants with a complete set of variables for regression analyses of asthma (AS), or asthma/hayfever (AS/HF), along with dietary habits and relevant covariates, out of a total baseline sample of 266,848. Participants completed a baseline questionnaire and provided self-reported information about their background, lifestyle, health, and health service. A written informed consent was obtained from all the participants and the ethics approval for the 45 and Up Study was provided by the University of Sydney Human Research Ethics Committee. More detailed information about the 45 and Up Study can be found elsewhere [41]. Approval to use the data for the present study was obtained by agreement between The University of Western Sydney and The Sax Institute.

All included variables were examined by using self-reported data from the 45 and Up Study baseline questionnaire. Body mass index (BMI: weight in kg/height in m^2^) was determined from self-reported height and weight, categorized as underweight (BMI = ≤ 18.5), normal (BMI = 18.5–24.9), overweight (BMI = 25.0–29.9) or obese (BMI ≥ 30) according to World Health Organization classifications [42]. Physical activity was assessed via The Active Australia Survey, wherein participants self-reported minutes spent walking or doing moderate or vigorous physical activities over the previous week [43]. Demographic variables included age, sex, education level, smoking history (ever or never a regular smoker), Australian ancestry (or not), speak a language other than English at home (or not), marital status, and work status.

### Exposures: dietary habits

Dietary habits were examined with twelve questionnaire items that assessed frequency of consumption. Items assessing meats (beef, lamb, pork), poultry (chicken, turkey, duck), processed meats (bacon, sausages, salami, devon, burgers), fish or seafood, and cheese were based on the number of times eaten each week in all meals and snacks. Pieces or slices of brown or whole-meal bread (including multigrain, rye) and bowls of breakfast cereal were assessed based on the number usually eaten each week. Cooked and raw vegetables (including salad), fruits, and fruit juice were assessed based on the number of serves usually eaten per day. Alcoholic drinks (one drink = a glass of wine, middy of beer or nip of spirits) were assessed based on number had each week. Details of the questions can be found online at http://www.45andUp.org.au.

### Outcomes: asthma (AS) and asthma/hayfever (AS/HF)

For 20,376 of our included participants – those who completed an earlier version of the baseline questionnaire – the AS/HF variable was assessed by a dichotomous question: “Has a doctor ever told you that you have… asthma or hayfever?” Responses were coded as “yes” or “no.” For the remaining 135,659 of included participants, asthma and hayfever were assessed separately, but in analogous manner to the above dichotomous question. The separate asthma item was used in analyses specific to AS, or was combined with the hayfever item for analyses of AS/HF.

### Statistics

SPSS 19.0 (SPSS Inc., Chicago, IL) was used for descriptive and inferential statistics. Descriptive analyses included percentages, to provide characteristics of the sample. Mann–Whitney U was used to assess differences in characteristics by sex. To assess dietary factors [34], principle components analysis with Kaiser normalization and varimax rotation were used to produce a diverse and interpretable pattern of factor loadings [44]. Males and females were analyzed separately, due to differences in both dietary intake and prevalence of AS (See Tables 1 and 2). Factor retention was determined by Kaiser’s [45] criterion for un-rotated eigenvalues (>1.0), combined with factor loadings of at least 0.40 [46]. Factors underwent varimax orthogonal rotation to facilitate interpretability and minimize the correlation between factors. After rotation, 11 dietary variables loaded uniquely onto one of four dietary factors for men (meats/cheese; fruits/vegetables; poultry/seafood; grains/alcohol) or five dietary factors for women (meats; fruits/vegetables; poultry/seafood; cereal/alcohol; cheese/brown bread), accounting for 51% and 59% of the variance in measured dietary habits, respectively. Table 3 presents factor loadings for the identified dietary factors. All dietary variables loaded positively onto their respective factors, except for alcohol, which loaded negatively. Fruit juice failed to load on a dietary factor, likely due to very low consumption levels, and so was not used in further analyses.

**Table 1:** Characteristics of 45 and up study sample, new south wales, Australia, 2006-2009

		**Total sample**		**Males**		**Females**	
		**N = 156,035**		**n = 70,979**		**n = 85,056**	
**Variable**	**Subcategory**	**n**	**%**	**n**	**%**	**n**	**%**
Asthma/Hayfever history	Yes	35,571	22.8	13,289	18.7	22,282	26.2
	No	120,464	77.2	57,690	81.3	62,774	73.8
Age category	45-64 years	105,369	67.5	44,681	62.9	60,688	71.4
	65-74 years	31,579	20.2	16,000	22.5	15,579	18.3
	75-84 years	16,137	10.3	8,952	12.6	7,185	8.4
	85+ years	2,950	1.9	1,346	1.9	1,604	1.9
Education	No certificate or other qualifications	14,260	9.1	6,048	8.5	8,212	9.7
	School or intermediate certificate	32,334	20.7	9,835	13.9	22,499	26.4
	Higher school or leaving certificate	15,494	9.9	7,004	9.9	8,490	10.0
	Trade/apprenticeship	16,134	10.3	12,682	17.9	3,452	4.1
	Certificate/diploma	35,444	22.7	14,678	20.7	20,766	24.4
	University degree or higher	42,411	27.2	20,732	29.2	21,679	25.5
Weight status from body mass index	Underweight	1,855	1.2	457	0.6	1,398	1.6
	Normal	57,411	36.8	21,232	29.9	36,179	42.5
	Overweight	61,952	39.7	33,921	47.8	28,031	33.0
	Obese	34,817	22.3	15,369	21.7	19,448	22.9
Smoking history	Ever smoker	65,384	41.9	35,206	49.6	30,178	35.5
	Never smoker	90,651	58.1	35,773	50.4	54,878	64.5
Met 2 fruits + 5 vegetable standard	Yes	39,255	25.2	12,442	17.5	26,813	31.5
	No	116,780	74.8	58,537	82.5	58,243	68.5
Australian Ancestry	Yes	80,030	51.3	36,288	51.1	43,742	51.4
	No	76,005	48.7	34,691	48.9	41,314	48.6
Speak a language other than English in the home	Yes	13,370	8.6	6,505	9.2	6,865	8.1
	No	142,665	91.4	64,474	90.8	78,191	91.9
Marital status	Married, cohabitating, de facto	120,983	77.5	58,872	82.9	62,111	73.0
	Other	35,052	22.5	12,107	17.1	22,945	27.0
Work status	Work full time	41,838	26.8	22,958	32.3	18,880	22.2
	Retired	54,064	34.6	25,603	36.1	28,461	33.5
	Other	60,133	38.5	22,418	31.6	37,715	44.3

**Table 2:** Demographic and dietary characteristics by sex in the 45 and up study sample, new south wales, Australia, 2006-2009

	**Males**			**Females**		
	**n = 70,979**			**n = 85,056**		
**Variable**	**Mean**	**SD**	**Median frequency**	**Mean**	**SD**	**Median frequency**
Age (years)	62.2	10.6	–	60.2	10.2	–
Body mass index (kg/m2)	27.3	4.2	–	26.7	5.3	–
Red meat, times eaten each week	3.6	2.5	3	3.1	2.0	3
Poultry, times eaten each week	2.3	1.6	2	2.3	1.5	2
Processed meat, times eaten each week	1.8	1.8	1	1.2	1.4	2
Fish or seafood, times eaten each week	1.8	1.4	3	1.9	1.5	2
Cheese, times eaten each week	3.4	2.5	3	3.5	2.4	3
Brown/whole-meal bread, slices each week	11.8	10.4	10	9.5	7.3	8
Breakfast cereal, bowls each week	4.8	2.8	6	4.6	2.8	5
Cooked vegetables, serves each day	2.3	1.7	2	2.7	1.6	3
Raw vegetables, serves each day	1.3	1.3	1	1.7	1.4	1
Fruits, serves each day	1.9	1.4	2	2.1	1.3	2
Fruit juice, serves each day	0.8	1.1	1	0.7	1.0	0
Alcoholic drinks, number each week	10.0	11.7	7	4.7	6.2	2

Note: based on Mann–Whitney tests, all means differ significantly by sex, p < 0.05.

**Table 3:** Food item factor loadings from the rotated component matrix in women and men

	**Rotated factor loadings: men**				**Rotated factor loadings: women**				
**Foods**	**1**	**2**	**3**	**4**	**1**	**2**	**3**	**4**	**5**
Red meat, times eaten each week		.613				.772			
Processed meat, times eaten each week		.702				.676			
Cheese, times eaten each week		.652							.689
Brown/whole-meal bread, slices each week			.561						.729
Alcoholic drinks, number each week			-.576					-.727	
Breakfast cereal, bowls each week			.684					.685	
Poultry, times eaten each week				.735			.710		
Fish or seafood, times eaten each week				.776			.778		
Cooked vegetables, serves each day	.809				.804				
Raw vegetables, serves each day	.800				.797				
Fruits, serves each day	.556				.503				
**Factor variance explained (%)**	14.9	13.8	11.7	11.0	15.2	13.3	10.8	9.9	9.1
**Total variance explained (%)**				51.4					58.4

For each sex, two binary hierarchical logistic regression analyses were run to examine dietary influences on AS and on AS/HF. Hierarchical logistic regression models were structured with two blocks of variables entered simultaneously. Block one included all dietary factors to assess the unadjusted odds ratios of AS or AS/HF diagnosis. Block two adjusted for categories of age, education, physical activity, BMI, and smoking history. For all odds ratios, 95% confidence intervals were calculated, and a significance level of 0.05 was used for all analyses.

## Results

Table 1 presents the characteristics of our study sample. Almost 23% of participants reported ever having been diagnosed with AS/HF (males 18.7%; females 26.2%). Among participants with data on AS alone (data not shown in table), prevalence was lower (males 10.0%; females 13.7%). Table 2 presents differences in age, BMI, and dietary habits by sex, all statistically significant (*P* < 0.001).

Binary logistic regression analyses, used to investigate the relationship between dietary factors and AS and AS/HF for men, are presented in Table 4. Parallel analyses for women are presented in Table 5. For both tables, model 1 presents the unadjusted odds ratios (though each dietary factor is presented when controlling for all other dietary factors) with 95% confidence intervals (95%CI) for all dietary factors (though each dietary factor is presented when controlling for all other dietary factors). Model 2 presents the adjusted odds ratios with 95%CI, controlling additionally for age group and education category, smoking, physical activity category, and weight status.

**Table 4:** Binary logistic regression analysis: odds of men ever having been diagnosed with asthma or asthma hayfever by dietary factor in the 45 and up study sample, new south wales, Australia, 2006-2009

				**Asthma (n = 61,968)**		**Asthma/Hayfever (n = 70,979)**	
**Dietary factor**	**Mean age (years)**	**Ever smoker (%)**	**Mean BMI**	**Model 1**	**Model 2**	**Model 1**	**Model 2**
				**Unadjusted OR (95% CI)**	**Adjusted**^**#**^**OR (95% CI)**	**Unadjusted OR (95% CI)**	**Adjusted**^**#**^**OR (95% CI)**
**Fruits/vegetables**							
1^st^ quintile (reference)	61.02	50	27.31	1.00	1.00	1.00	1.00
2^nd^ quintile	61.72	49	27.29	0.97 (0.90 to 1.06)	0.98 (0.90 to 1.06)	1.01 (0.95 to1.07)	1.00 (0.94 to 1.06)
3^rd^ quintile	62.04	50	27.16	1.03 (0.95 to 1.11)	1.03 (0.95 to 1.12)	1.07 (1.00 to 1.13) *	1.06 (0.99 to 1.12)
4^th^ quintile	63.01	50	27.21	0.96 (0.89 to 1.05)	0.97 (0.90 to 1.06)	1.05 (0.99 to 1.12)	1.05 (0.99 to 1.12)
5^th^ quintile	64.22	50	27.37	0.94 (0.86 to 1.02)	0.97 (0.89 to 1.05)	0.95 (0.89 to 1.01)	0.98 (0.92 to 1.04)
*p* value for trend	–	–	–	0.257	0.557	0.001	0.034
**Meats/cheese**							
1^st^ quintile (reference)	61.97	43	26.72	1.00	1.00	1.00	1.00
2^nd^ quintile	62.41	48	27.22	1.00 (0.92 to 1.09)	1.01 (0.93 to 1.10)	1.04 (0.98 to 1.11)	1.06 (0.99 to 1.12)
3^rd^ quintile	62.49	49	27.24	1.02 (0.94 to 1.11)	1.04 (0.95 to 1.13)	1.06 (1.00 to 1.13) *	1.09 (1.02 to 1.15) *
4^th^ quintile	62.78	53	27.42	1.06 (0.97 to 1.15)	1.08 (0.99 to 1.17)	1.12 (1.05 to 1.19) *	1.15 (1.08 to 1.22) *
5^th^ quintile	62.35	56	27.75	1.15 (1.06 to 1.25) *	1.18 (1.08 to 1.28) *	1.17 (1.10 to 1.24) *	1.22 (1.14 to 1.29) *
*p* value for trend	–	–	–	0.004	0.001	< 0.001	< 0.001
**Poultry/seafood**							
1^st^ quintile (reference)	63.26	53	27.03	1.00	1.00	1.00	1.00
2^nd^ quintile	62.51	51	27.27	0.98 (0.91 to 1.07)	0.98 (0.90 to 1.07)	1.02 (0.96 to 1.08)	1.01 (0.95 to 1.07)
3^rd^ quintile	62.45	49	27.28	0.98 (0.90 to 1.06)	0.97 (0.89 to 1.06)	1.03 (0.97 to 1.09)	1.01 (0.95 to 1.07)
4^th^ quintile	62.26	48	27.36	1.00 (0.92 to 1.09)	1.00 (0.92 to 1.09)	1.09 (1.02 to 1.15) *	1.06 (1.00 to 1.13) *
5^th^ quintile	61.52	48	27.39	1.09 (1.01 to 1.18) *	1.08 (0.99 to 1.17)	1.14 (1.08 to 1.21) *	1.11 (1.04 to 1.17) *
*p* value for trend	–	–	–	0.055	0.102	< 0.001	0.002
**Grains/alcohol**							
1^st^ quintile (reference)	59.50	63	27.85	1.00	1.00	1.00	1.00
2^nd^ quintile	60.94	53	27.70	1.02 (0.94 to 1.11)	1.03 (0.95 to 1.12)	1.06 (1.00 to 1.12)	1.05 (0.99 to 1.11)
3^rd^ quintile	62.55	47	27.29	0.95 (0.87 to 1.03)	0.97 (0.89 to 1.05)	1.03 (0.97 to 1.09)	1.02 (0.96 to 1.08)
4^th^ quintile	64.12	44	27.00	1.03 (0.94 to 1.11)	1.05 (0.97 to 1.14)	1.07 (1.00 to 1.13) *	1.06 (1.00 to 1.13) *
5^th^ quintile	64.88	41	26.51	0.93 (0.85 to 1.01)	0.96 (0.88 to 1.04)	1.02 (0.96 to 1.08)	1.02 (0.96 to 1.09)
*p* value for trend	–	–	–	0.057	0.137	0.195	0.277

Notes: OR = odds ratio; BMI = body mass index in kg/m^2^; CI = confidence interval; *Significantly different from reference category, *P* < 0.05; ^#^Model adjusted for age group (45–64, 65–74, 75–84, 85+), education (no school certificate, school or intermediate certificate, high school or leaving certificate, trade apprentice, certificate or diploma, university degree or higher), weight status (normal, underweight, overweight, obese), physical activity weekly minutes quartile, and smoking status (never smoked, ever smoked).

**Table 5:** Binary logistic regression analysis: odds of women ever having been diagnosed with asthma or asthma/hayfever by dietary factor in the 45 and up study sample, new south wales, Australia, 2006-2009

				**Asthma (n = 73,691)**		**Asthma/Hayfever (n = 85,056)**	
**Dietary factor**	**Mean age (years)**	**Ever smoker (%)**	**Mean BMI**	**Model 1**	**Model 2**	**Model 1**	**Model 2**
				**Unadjusted OR (95% CI)**	**Adjusted**^**#**^**OR (95% CI)**	**Unadjusted OR (95% CI)**	**Adjusted**^**#**^**OR (95% CI)**
**Fruits/vegetables**							
1^st^ quintile (reference)	59.29	39	26.72	1.00	1.00	1.00	1.00
2^nd^ quintile	59.51	35	26.57	0.99 (0.92 to 1.06)	0.99 (0.92 to 1.06)	1.05 (1.00 to 1.10)	1.03 (0.98 to 1.08)
3^rd^ quintile	60.42	34	26.68	1.00 (0.94 to 1.07)	1.00 (0.93 to 1.07)	1.08 (1.03 to 1.13) *	1.05 (1.00 to 1.10)
4^th^ quintile	61.14	34	26.76	1.05 (0.98 to 1.12)	1.04 (0.98 to 1.12)	1.11 (1.05 to 1.16) *	1.09 (1.03 to 1.14) *
5^th^ quintile	61.82	34	26.81	1.04 (0.97 to 1.11)	1.05 (0.98 to 1.12)	1.07 (1.02 to 1.12) *	1.07 (1.02 to 1.12) *
*p* value for trend	–	–	–	0.384	0.259	0.001	0.011
**Meats**							
1^st^ quintile (reference)	60.25	37	25.53	1.00	1.00	1.00	1.00
2^nd^ quintile	60.48	36	26.46	1.01 (0.94 to 1.08)	0.99 (0.92 to 1.06)	1.05 (1.00 to 1.11) *	1.05 (1.00 to 1.11) *
3^rd^ quintile	60.31	35	26.86	1.07 (1.00 to 1.14)	1.03 (0.96 to 1.10)	1.14 (1.08 to 1.19) *	1.13 (1.07 to 1.18) *
4^th^ quintile	60.37	35	27.18	1.10 (1.03 to 1.17) *	1.04 (0.97 to 1.12)	1.19 (1.13 to 1.25) *	1.17 (1.12 to 1.23) *
5^th^ quintile	60.77	35	27.55	1.12 (1.05 to 1.19) *	1.06 (0.99 to 1.13)	1.26 (1.20 to 1.32) *	1.25 (1.19 to 1.31) *
*p* value for trend	–	–	–	0.002	0.253	< 0.001	< 0.0001
**Poultry/seafood**							
1^st^ quintile (reference)	61.49	36	26.31	1.00	1.00	1.00	1.00
2^nd^ quintile	60.32	35	26.52	0.97 (0.91 to 1.04)	0.96 (0.90 to 1.03)	1.01 (0.96 to 1.06)	0.99 (0.94 to 1.04)
3^rd^ quintile	60.34	35	26.63	0.99 (0.93 to 1.06)	0.97 (0.91 to 1.04)	1.01 (0.96 to 1.06)	0.99 (0.94 to 1.04)
4^th^ quintile	60.11	35	26.82	0.99 (0.92 to 1.06)	0.96 (0.90 to 1.02)	1.04 (0.99 to 1.09)	1.01 (0.96 to 1.06)
5^th^ quintile	59.92	36	27.27	1.12 (1.05 to 1.19) *	1.06 (1.00 to 1.14)	1.12 (1.06 to 1.17) *	1.06 (1.01 to 1.12) *
*p* value for trend	–	–	–	< 0.001	0.011	0.0001	0.016
**Cereal/alcohol**							
1^st^ quintile (reference)	57.64	53	26.34	1.00	1.00	1.00	1.00
2^nd^ quintile	58.76	39	26.84	0.98 (0.92 to 1.05)	0.99 (0.93 to 1.06)	0.99 (0.95 to 1.04)	1.00 (0.95 to 1.04)
3^rd^ quintile	59.72	33	26.77	0.93 (0.87 to 0.99) *	0.95 (0.89 to 1.02)	0.97 (0.93 to 1.02)	0.98 (0.93 to 1.03)
4^th^ quintile	61.59	29	26.77	0.97 (0.90 to 1.03)	1.00 (0.94 to 1.07)	1.01 (0.96 to 1.06)	1.03 (0.98 to 1.08)
5^th^ quintile	64.47	23	26.85	0.99 (0.93 to 1.06)	1.05 (0.98 to 1.12)	1.02 (0.98 to 1.07)	1.07 (1.02 to 1.13) *
*p* value for trend	–	–	–	0.199	0.104	0.346	0.005
**Cheese/brown bread**							
1^st^ quintile (reference)	58.24	35	27.02	1.00	1.00	1.00	1.00
2^nd^ quintile	59.29	36	26.84	0.92 (0.86 to 0.98) *	0.92 (0.86 to 0.98) *	0.94 (0.90 to 0.99) *	0.94 (0.90 to 0.99) *
3^rd^ quintile	60.37	35	26.78	0.87 (0.82 to 0.93) *	0.88 (0.83 to 0.94) *	0.95 (0.90 to 0.99) *	0.95 (0.90 to 1.00) *
4^th^ quintile	61.43	35	26.54	0.88 (0.82 to 0.94) *	0.89 (0.84 to 0.96) *	0.95 (0.91 to 1.00) *	0.96 (0.92 to 1.01)
5^th^ quintile	62.84	36	26.39	0.86 (0.80 to 0.91) *	0.88 (0.82 to 0.94) *	0.95 (0.90 to 0.99) *	0.97 (0.92 to 1.02)
*p* value for trend	–	–	–	< 0.001	< 0.001	0.090	0.136

Notes: OR = odds ratio; BMI = body mass index in kg/m^2^; CI = confidence interval; *Significantly different from reference category, *P* < 0.05; ^#^Model adjusted for age group (45–64, 65–74, 75–84, 85+), education (no school certificate, school or intermediate certificate, high school or leaving certificate, trade apprentice, certificate or diploma, university degree or higher), weight status (normal, underweight, overweight, obese), physical activity weekly minutes quartile, and smoking status (never smoked, ever smoked).

### Association with dietary factors: men

For diagnosed asthma, there was a positive association with the meats/cheese dietary factor in the unadjusted model (OR for the highest versus lowest quintile = 1.15, 95%CI = 1.06, 1.25; *P*_trend_ = 0.004). In the model that adjusted for relevant covariates, this monotonic association was strengthened (AOR for the highest versus lowest quintile = 1.18, 95%CI = 1.08, 1.28; *P*_trend_ = 0.001). Similarly, for diagnosed AS/HF, the meats/cheese dietary factor was positively and monotonically associated in the unadjusted model (OR for the highest versus lowest quintile = 1.17, 95%CI = 1.10, 1.24; *P*_trend_ < 0.001). In the adjusted model, this monotonic association was also strengthened (AOR for the highest versus lowest quintile = 1.22, 95%CI = 1.14, 1.29; *P*_trend_ < 0.001). Thus, the meats/cheese dietary factor was consistently and monotonically positively related to diagnosis of AS and AS/HF in our sample of men.

For the poultry/seafood dietary factor there was a weak positive association with AS that did not reach statistical significance in the unadjusted model (OR for the highest versus lowest quintile = 1.09, 95%CI = 1.01, 1.18; *P*_trend_ = 0.055). This association was not significant in the model that adjusted for relevant covariates (AOR for the highest versus lowest quintile = 1.08, 95%CI = 0.99, 1.17; *P*_trend_ = 0.102). In contrast, for diagnosed AS/HF, the poultry/seafood dietary factor was positively associated in the unadjusted model (OR for the highest versus lowest quintile = 1.14, 95%CI = 1.08, 1.21; *P*_trend_ < 0.001). In the adjusted model, this association was attenuated slightly (AOR for the highest versus lowest quintile = 1.11, 95%CI = 1.04, 1.17; *P*_trend_ = 0.002). In sum, the poultry/seafood dietary factor showed positive monotonic relationships with AS/HF diagnosis, but was inconstantly related to AS diagnosis in these men.

The fruits and vegetables factor showed a weak inconstant protective relationship in the unadjusted model (OR for the highest versus lowest quintile = 0.95, 95%CI = 0.89, 1.01; *P*_trend_ = 0.001), but adjusting for covariates attenuated the relationship (AOR = 0.98, 95%CI = 0.92, 1.04; *P*_trend_ = 0.034). All other dietary factors were not significant influences on diagnosed asthma, or on diagnosed AS/HF.

### Association with dietary factors: women

For diagnosed asthma, there was an inverse association with the cheese/brown bread dietary factor in the unadjusted model (OR for the highest versus lowest quintile = 0.86, 95%CI = 0.80, 0.91; *P*_trend_ < 0.001). Adjusting for relevant covariates left this pattern of association nearly identical to the unadjusted model (AOR for the highest versus lowest quintile = 0.88, 95%CI = 0.82, 0.94; *P*_trend_ < 0.001). For diagnosed AS/HF, the cheese/brown bread factor showed a weak protective association such that all other levels of intake above the lowest quintile had odds ratios below unity, but the linear trend was not significant (OR for the highest versus lowest quintile = 0.95, 95%CI = 0.90, 0.99; *P*_trend_ = 0.090). This pattern of relationships was attenuated in the adjusted model (AOR for the highest versus lowest quintile = 0.97, 95%CI = 0.92, 1.02; *P*_trend_ = 0.136). Thus, the cheese/brown bread factor was a consistent protective factor for asthma, but less consistently protective for AS/HF in our sample.

For diagnosed asthma, there was a positive monotonic association with the dietary meats factor in the unadjusted model (OR for the highest versus lowest quintile = 1.12, 95%CI = 1.05, 1.19; *P*_trend_ = 0.002). In the model that adjusted for relevant covariates, this association was attenuated and not significant (AOR for the highest versus lowest quintile = 1.06, 95%CI = 0.99, 1.13; *P*_trend_ = 0.253). For diagnosed AS/HF, the meats factor was positively and monotonically associated in the unadjusted model (OR for the highest versus lowest quintile = 1.26, 95%CI = 1.20, 1.32; *P*_trend_ < 0.001), and this relationship showed the same pattern in the adjusted model (AOR for the highest versus lowest quintile = 1.25, 95%CI = 1.19, 1.31; *P*_trend_ = 0.001). Thus, the meats factor was a risk factor in three of the four models for women.

There was a significant association between the poultry/seafood factor and diagnosed AS in the unadjusted model (OR for the highest versus lowest quintile = 1.12, 95%CI = 1.05, 1.19; *P*_trend_ < 0.001). In the model that adjusted for relevant covariates, this association was attenuated but remained significant (AOR for the highest versus lowest quintile = 1.06, 95%CI = 1.00, 1.14; *P*_trend_ = 0.011). For diagnosed AS/HF, the poultry/seafood factor was positively associated in the unadjusted model (OR for the highest versus lowest quintile = 1.12, 95%CI = 1.06, 1.17; *P*_trend_ = 0.001). This association was also attenuated, but remained significant when controlling for relevant covariates (AOR for the highest versus lowest quintile = 1.06, 95%CI = 1.01, 1.12; *P*_trend_ = 0.016). The poultry/seafood dietary factor was shown to be a significant risk factor in all four models.

The fruits and vegetables dietary factor showed no significant relationships with diagnosed asthma, but appeared to be a risk factor for AS/HF diagnosis (OR for the highest versus lowest quintile = 1.07, 95%CI = 1.02, 1.12; *P*_trend_ = 0.001). In this unadjusted model, the top three quintiles of fruits/vegetables intake showed higher odds of diagnosis, in comparison to the lowest consumption level. After adjusting for covariates, the patterns remained similar to the unadjusted model (AOR for the highest versus lowest quintile = 1.07, 95%CI = 1.02, 1.12; *P*_trend_ = 0.011).

The cereal/alcohol dietary factor showed little relationship with diagnosed AS in either model. For AS/HF diagnosis, however, there was an inconstant but significant linear relationship in the adjusted model (AOR for the highest versus lowest quintile = 1.07, 95%CI = 1.02, 1.13; *P*_trend_ = 0.005). All other dietary factors were not significant influences on AS/HF.

## Discussion

Results showed prevalence of asthma/hayfever (AS/HF) among the participants in this large cohort was 19-26% and prevalence of asthma (AS) was 10-14%, with higher prevalence in women, and these findings correspond closely with National Health Survey data from a similar time period [2]. The main finding of this cross-sectional study, however, was that dietary factors are associated with diagnosed AS and AS/HF in Australian adults of middle age or older. In looking at the analyses for both sexes, diets generally high in meat, particularly diets marked by greater consumption of poultry, seafood, and red and processed meats in females, and diets marked by greater amounts of red meat, processed meat, and cheese consumption in males, appear to be risk factors for AS and AS/HF diagnosis in this population. In comparisons of those in the highest category of these dietary factors to the lowest respective categories, most of the analyses showed increased odds in the 10-25% range. Additionally, the cheese/brown bread factor was shown to be a protective for AS diagnosis in women. Taken together, the overall pattern of these risk and protective factors suggests that adherence to a more meat-based diet may pose risk for AS and AS/HF in Australian adults.

Our findings are supported in previous literature on diet and asthma risk in both children and adults [21,25,30,35,40], though adult studies on dietary patterns have been somewhat limited and equivocal to date [47]. In a large New Zealand children’s cohort study, eating hamburgers was associated with a higher lifetime prevalence of asthma and wheeze [25]. A high-meat diet, however, was not a risk factor for asthma or wheeze [25]. In a cross-sectional study of teenagers in Taiwan, consumption of liver, butcher’s meat, and fried foods were associated with increased risk of asthma, and liver also was shown to be a risk factor for allergic rhinitis [23].

In the adult literature, several studies were in agreement with the primary findings of the current study [35,38,40]. Although the current study did not examine Mediterranean, Cosmopolitan, or Prudent dietary patterns previously reported in adult literature on diet and asthma, our results support studies that suggest a contribution of a Western diet (characterized by high intakes of red meat, saturated fat, and refined grains, combined with low intakes of whole grains) to impaired lung function, increased asthma prevalence and worsened asthma control [35,40].

Our findings failed to support, however, studies suggesting that a diet high in fruits and vegetables may have a protective association with allergic rhinitis [38] and asthma in adulthood [33,39]. In contrast, we found weak and inconsistent associations for the fruits/vegetables factor in men, and significant positive relationships with AS/HF in women. The reasons underlying this surprising finding are unclear, but there are several possibilities. Our dietary measure was admittedly crude, and we had no way of differentiating frequently consumed high-fat versions of vegetables such as hot chips, other fried vegetables, or those mixed in casseroles and meat pies. Previous studies have shown deep-fried or high-fat foods may be a risk factor for asthma [21,23,48,49]. Also, total energy intake was not assessed, and this could contribute to potential confounding in analyses. Reverse causality is also a possibility [50], such that those diagnosed with AS/HF might purposefully eat more fruits and vegetables in efforts to bolster health and control symptoms or attacks. Lastly, there are a number of fruits and vegetables that are high in histamine or other biogenic amines, and histamine intolerance may play a role in the observed relationship with fruits and vegetables. The common symptoms of histamine intolerance include sneezing, congestion, rhinorrhea, and asthma [51]. In some individuals, these reactions can occur even after small amounts of histamine are ingested [51].

Our study findings are contrary to studies that have shown no relationship between Western dietary patterns or “vegetarian” dietary patterns and asthma [30,36], possibly due to differing populations and methods of dietary assessment. Lastly, our results did not agree with an observed protection associated with fish or seafood intake [16]. In contrast, our study found that fish or seafood was linked with poultry consumption, and together this factor posed a risk for AS and AS/HF in the majority of analyses, which supports earlier work from the Netherlands [35].

### Potential mechanisms

Generally, Australians eat a calorically dense and nutrient-poor Western diet [52,53]. A typical Western diet is low in antioxidants, high in fat and saturated fat, and provides a surplus of calories [54], any of which could contribute to increased asthma prevalence by modulating the innate immune response [54]. Among potential factors to consider in determining how a Western diet, or other meat-based diets, may pose a risk for asthma and/or hayfever are saturated fat consumption, exposures from processing or cooking, and fast food and takeaway consumption, marked by high intakes of sodium and fat.

The calorically dense, nutrient poor diet consumed by many Australians can result in post-prandial dysmetabolism and increased oxidative stress. The transient increase in free radicals may acutely trigger inflammation and endothelial dysfunction [55]. Consumption of meat, particularly red meat, has been associated with inflammation and oxidative stress, which may partly explain the association between meat consumption and chronic disease risk [37,56]. Also, fat content, including saturated fat in red meat, and dietary cholesterol may play an important role in the link to chronic disease [57]. A high fat intake and plasma triglyceride levels have been associated with airway hyper-responsiveness, asthma risk, and adult-onset wheeze [54]. High-fat meals have been shown to increase neutrophilic airway inflammation, and the effect is determined by the type of fat consumed [48,58]. High-fat meals also increase triglycerides and exhaled nitric oxide, a marker of airway inflammation, two-hours post-prandially, and suppress bronchodilator recovery in asthma [49,54].

Processed meats contain mutagens, including N-nitroso compounds and heterocyclic amines (HCAs), and polycyclic aromatic hydrocarbons (PAHs) formed during high-temperature cooking and grilling [59]. Polycyclic aromatic hydrocarbons, in particular, have been linked to respiratory disorders and have been shown to trigger asthma attacks in asthmatics [60]. Eating meats cooked at higher temperatures, such as barbecues and grilling, also increases the formation of PAHs [59]. Previous studies have shown blood levels of PAHs are significantly correlated with grilled and smoked meat intake among asthmatic children [61].

High-meat diets may be linked with fast food or takeaway consumption, typically high in sodium and saturated fat, and implicated in asthma [53,62,63]. The issue of risk from saturated fat poses an explanatory problem with regard to cheese, however, as cheese loaded on a protective factor with brown and whole-meal bread for females, but on a risk factor with red and processed meats for males. It is possible that cheese is a marker of a more prudent diet for females, due to concerns regarding calcium intake and osteoporosis prevention. It is also possible that women consumed fat-free or low-fat cheeses that would not have been high in saturated fat. There is evidence, however, from a pediatric prospective cohort study that both brown bread and milk fat are protective against asthma symptoms [64].

### Strengths and limitations

Our findings should be viewed in light of a number of limitations, and foremost among these is the cross-sectional study design, which allows only the establishment of associations between variables, not temporal sequence or causal direction. In our study, we did not have access to information on relevant comorbidities, including anemia, cardiac dysfunction, systemic inflammation, gastro-esophageal reflux disease, sleep apnea, or chronic obstructive pulmonary disorder. Thus, we cannot rule out potential confounding associated with these comorbid conditions.

Selection bias is a potential concern for any study relying on voluntary participation from the population, and the 18% response rate obtained in The 45 and Up Study, combined with exclusion of participants without a full set of data in the current study warrants caution with regard to external validity. It is conceivable that the 156,035 participants in the current study could differ from non-participants in potentially meaningful ways. The 45 and Up Study, however, is the largest study of healthy aging to be carried out in the Southern Hemisphere, and is likely to be one of the more representative large-scale cohort studies conducted globally.

Self-report instruments are subject to a number of potential biases, and misclassifications of both exposure and outcome could result. In particular, our available measures of dietary habits were limited in scope, and generally did not provide information on portion size or other relevant attributes of foods consumed beyond the frequency of consumption. As such, we could not control for energy intake, although our covariates of body mass index and physical activity categories may have partially accounted for this potential influence.

Counterbalancing these limitations were several strengths. The large and diverse sample of the adult and older adult population of Australia’s most populous state allowed us to examine a group not often studied with regard to diet and asthma. Our study analyzed males and females separately, allowing us to establish dietary factors unique for each sex, and to assess the independent relationships between those factors and our outcomes of interest. Furthermore, we controlled for several potential key confounders: physical activity, smoking, age, education, and body mass index. Lastly, we were able to test and confirm our findings for relationships between diet and AS/HF in a large subset of participants with data on asthma, separated out from hayfever.

### Future directions

Future studies should include analysis of dietary patterns and asthma and hayfever over time, as longitudinal data become available from the 45 and Up study. It may be worthwhile to examine a subsample of this cohort using more rigorous measures of dietary intake, asthma, and hayfever. Additional epidemiological studies with more rigorous measures of exposures and outcomes should be undertaken in populations of differing ages, ethnicities, geographical locations, and in those with various co-morbidities. Lastly, dietary interactions with other environmental and genetic influences should be investigated with prospective designs.

## Conclusions

From our data, there is evidence of increased risk of asthma diagnosis, and risk of asthma/hayfever diagnosis, in association with adherence to a diet with higher meat consumption. Future studies are needed to examine and explain dietary influences on asthma and asthma/hayfever in adult populations, possibly through integration of epidemiological findings with clinical studies or basic science. Given the relatively high prevalence of asthma and hayfever in Western countries, findings from studies of diet and airway health may be useful in reducing the burden of these conditions through changes in dietary habits.

## Abbreviations

AOR: Adjusted odds ratio; AS: Asthma; AS/HF: Asthma or hayfever; BMI: Body mass index; CI: Confidence interval; HCAs: Heterocyclic amines; OR: Odds ratio; PAHs: Polycyclic aromatic hydrocarbons.

## Competing interests

The authors have no financial competing interests to declare, but note that one of the authors is a vegetarian.

## Authors’ contributions

RR conceived the study, led statistical analyses and manuscript writing, and took responsibility for quality assurance and control. SR assisted in planning the study and conducting statistical analyses, and contributed to manuscript writing. KN conducted the literature review and contributed to manuscript writing. All authors approved the submitted manuscript.

## Authors’ information

RR and SR both earned PhD degrees in Human Nutrition, and are now assistant professors in the Department of Human Nutrition at Kansas State University in the USA. RR is an adjunct fellow at the University of Western Sydney in Australia, where the majority of work from this manuscript was completed. KN recently completed an internship with University of Western Sydney and earned a MS degree in Human Nutrition from Wageningen University in The Netherlands.
